# Toxicological Evaluation, Ultrasound‐Assisted Phytochemicals, Antioxidant, GC–MS, FTIR, and In Vivo Analysis of *Hibiscus rosa‐sinensis* Flowers

**DOI:** 10.1002/fsn3.71299

**Published:** 2025-11-30

**Authors:** Hassan Raza, Muhammad Tauseef Sultan, Ahmad Mujtaba Noman, Ali Imran, Sheraz Ahmed Bhatti, Iqra Baig, Muhammad Abdul Rahim, Meshari A. Alsuwat, Mohamed Fawzy Ramadan, Eliasse Zongo

**Affiliations:** ^1^ Department of Food Science & Technology, Faculty of Food Science and Nutrition Bahauddin Zakariya University Multan Pakistan; ^2^ Department of Human Nutrition, Faculty of Food Science and Nutrition Bahauddin Zakariya University Multan Pakistan; ^3^ Department of Food Science Government College University Faisalabad Faisalabad Pakistan; ^4^ Department of Pathobiology, Faculty of Veterinary Sciences Bahauddin Zakariya University Multan Pakistan; ^5^ Department of Food Science & Nutrition, Faculty of Medicine and Allied Health Sciences Times University Multan Pakistan; ^6^ Department of Basic Sciences, College of Nursing Taif University Taif Saudi Arabia; ^7^ Department of Clinical Nutrition, Faculty of Applied Medical Sciences Umm Al‐Qura University Makkah Saudi Arabia; ^8^ Laboratory of Research and Teaching in Animal Health and Biotechnology Nazi Boni University Bobo‐Dioulasso Burkina Faso

**Keywords:** FTIR, GC–MS, *Hibiscus rosa‐sinensis*, safety, UAE

## Abstract

The application of medicinal plants in the healthcare system is a centuries‐old practice, which is proving beneficial to communities. *Hibiscus rosa‐sinensis* , a flowering plant in the family Malvaceae, is used for both ornamental and medicinal purposes. The current research study evaluates ultrasound‐assisted extraction (UAE) of phytochemicals, antioxidant profiling, and safety of *Hibiscus rosa‐sinensis* powder and methanolic extracts in an animal model. Antioxidant samples were prepared in a ratio of 1:20, with the results showing the highest 2,2‐diphenyl‐1‐picrylhydrazyl (DPPH) and 2,2′‐azinobis (3‐ethylbenzothiazoline‐6‐sulfonic acid) (ABTS) activity in the methanol extract at 86.45% and 91.12%. Functional groups were examined using a Bruker Alpha FTIR spectrometer, and the analysis revealed the presence of O–H, –COOH, C≡N, C=O, and C–H functional groups. Gas Chromatography–Mass Spectrometry (GC–MS) profiling was done using a GC/MSD thermal desorption system, and results showed major compounds with the highest percentage are 1,2‐Cyclohexanedicarboxylic acid, bis (2‐ethylhexyl) ester 2‐[3‐Chlorophenoxy] propionamide (25.29%), and 1,2‐Cyclohexanedicarboxylic acid, bis (2‐ethylhexyl) ester, 2‐[3‐Chlorophenoxy] propionamide (17.77%), respectively. For the sub‐acute toxicity study, 25 male Sprague Dawley rats were distributed in five groups and supplemented with the following doses of flower powder and extracts (Control: Placebo, *Hibiscus rosa‐sinensis* powder diet 1 (HRPD1): 500 mg/kg BW powder, HRPD2: 1000 mg/kg BW powder, *Hibiscus rosa‐sinensis* extract diet 1 (HRED1): 100 mg/kg BW extract, and HRED2: 200 mg/kg extract) for 28 days. The safety evaluation showed no adverse impact and toxic signs in animals, as the values of hematological and biochemical parameters remained in normal ranges, with no hepato‐renal changes noticed in histopathological examination. In a nutshell, *Hibiscus rosa‐sinensis* exhibited significant bioactive compounds and functional groups, paving the way for its application in pharmaceuticals and nutraceuticals. Moreover, the powder and extracts proved safe at this dose; however, further preclinical trials are required at a high dose to validate their safety and efficacy in clinical trials.

## Graphical abstract

Phthalates and ethyl esters are dominant compounds, with O–H, –COOH, C≡N, C=O, and C–H as functional groups. Toxicological evaluations demonstrated that both powder and extract forms were well tolerated in animals, with no significant adverse effects observed in biochemical, hematological, or histopathological parameters. Although, slight changes were noted in the differential cell count at higher doses (1000 mg/kg powder and 200 mg/kg extract), these were not deemed harmful. Overall, the findings support the safe use of *H. rosa‐sinensis* as a supplement.

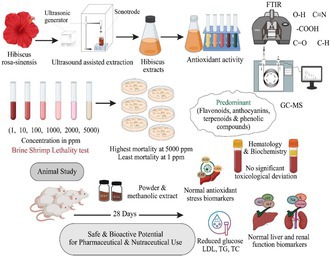

## 1 Introduction

Human health and well‐being face several challenges that require an inclusive and integrative approach to improve overall life quality. Medicinal plants and herbs play a vital role in healthcare due to their rich nutritional value and remarkable phytochemicals. Moreover, recent global trends have shifted towards plants and plant‐based products due to their natural and safe origin (Davis and Choisy 2024). The global herbal medicine market was valued at $233.08 billion USD in 2024. The market is projected to grow from $251.25 billion USD in 2025 to $437 billion USD by 2032, revealing a compound annual growth rate (CAGR) of 8.23% during the prediction period. European countries dominated the herbal medicine market with a market share of 44.55% in 2024 (Insights 2024). The medicinal herbs and their different parts treat common health problems like cough, fever, vomiting, diarrhea, and headache. Furthermore, numerous chronic metabolic conditions such as diabetes mellitus (DM), hypertension (HTN), cardiovascular diseases (CVDs), hepato‐renal syndrome (HRS), gastrointestinal (GI) ailments, cancer, and neurodegenerative issues can be effectively managed through herbal medicine (Arif et al. 2024). These therapeutic potentials are due to their bioactive compounds like terpenes, flavonoids, polyphenols, saponins, tannins, and carotenoids, which hold antioxidant and anti‐inflammatory activities (Dar et al. 2023).

*Hibiscus rosa‐sinensis* , commonly known as Chinese *Hibiscus* or China rose, is a tropical ornamental plant native to China but widely present in warm climates worldwide. The likely origin of the plant is tropical Asia, where it has been cultivated in China, Japan, and the Pacific Islands for a considerable period (Magdalita and San Pascual 2022). The nutritional profile of *Hibiscus rosa‐sinensis* significantly contributes to improving healthy lifestyles and overall well‐being. The leaves and flowers of *Hibiscus rosa‐sinensis* are rich in fiber, protein, Vitamin A, E, C, and B2, and minerals such as potassium, iron, calcium, phosphorus, sodium, magnesium, and manganese (Bala et al. 2022; Eze and Nwibo 2017). *Hibiscus rosa‐sinensis* is rich in bioactive compounds, including caffeic acid, gallic acid, p‐coumaric acid, quercetin, kaempferol, anthocyanins, and sterols (Amtaghri et al. 2024). These constituents contribute to a broad spectrum of pharmacological effects, including anti‐inflammatory, anticancer, antidiabetic, antihypertensive, hepatoprotective, renoprotective, and cardioprotective activities, enhancing their therapeutic potential (Rasul et al. 2023; Lu et al. 2022). Novel delivery systems such as nanoparticles (NPs) are an effective approach to enhance the hepatoprotective activity of *Hibiscus rosa‐sinensis* . In this context, various hepatic cell lines were treated with *H. rosa‐sinensis* Ag NPs to determine their oncoprotective effect against liver carcinoma (Lu et al. 2022).

Medicinal plants are crucial in healthcare, offering natural remedies for various diseases. Despite their prime significance, several people lack knowledge about medicinal herbs, phytochemistry, and their underlying mechanisms, leading to misuse or missed opportunities for natural healing. This research focuses on the phytochemical analysis of *H. rosa‐sinensis* using GC–MS and FTIR spectroscopy. The novelty of this paper lies in the UAE antioxidant and phytochemical profiling of *H. rosa‐sinensis* . Moreover, it also highlights toxicity assessment through the brine shrimp lethality assay and sub‐acute toxicity evaluation in an animal model, aiming to determine safe dosage levels and the potential safety of the plant flower powder and extract.

## 2 Materials and Methods

### 2.1 Plant Material

*Hibiscus rosa‐sinensis* flowers were collected from the vicinity of Multan (30.1864°N, 71.4886°E) and Layyah (30.9693°N, 70.9428°E) in April 2023. Plant flowers were identified by the botanist Prof. Dr. Zafar Ullah (Department of Botany, BZU, Multan, Pakistan). The plant identification number/voucher specimen number is www.wfo.org/taxon/wfo‐0000723007. All the reagents, chemicals and solvents that were used in the current study for antioxidants, phytochemical analysis, and in vivo study were of analytical grade.

### 2.2 Ultrasound‐Assisted Extraction (UAE) of *Hibiscus rosa‐sinensis* Extract

The flowers of *Hibiscus rosa‐sinensis* were gently washed and solar‐dried in a specifically designed solar drier with humidity and air regulator fitted inside to smooth the circulation of heat and air. The flowers were ground in an electric grinder (QE‐200, PC, Pamico Technologies). The resulting powder sample was mixed with solvents (acetone, methanol, distilled water, and hexane) for UAE at a 1:20 ratio (solid:liquid). The beaker with the solution was placed inside the ultrasonic chamber of a probe‐type ultrasonicator (VCX130, Sonics & Materials Inc., USA). UAE was operated at a 40 kHz frequency with 180 W of power output. An intensity probe was put inside the beaker for a time of 30 min (with 1 min on and off cycle to avoid buildup of heat). During the whole process, the temperature was maintained under 40°C to minimize loss of volatile compounds. After that, the mixture was filtered using 10‐μm filter paper. The filtrate was placed in a rotary evaporator (Heidolph Germany, REV‐2000B) for concentration at 45°C under vacuum conditions to procure a semi‐solid extract with 20.3% yield on a dry basis, and then removed to keep it till further use.

### 2.3 *Hibiscus rosa‐sinensis* Extract Analysis

#### 2.3.1 Total Phenolic Content (TPC)

In this study, the solvent extraction method was used, in which methanol, acetone, hexane, and distilled water were used as solvents, following the procedure of Silva et al. (2018) with minor changes. The TPC of *Hibiscus rosa‐sinensis* was determined by using 6 mL of Folin–Ciocalteau reagent mixed with 4% (1 mL) sodium carbonate solution and 0.5 mL of each extract. The solution was placed in the dark at room temperature for 90 min. Absorbance was measured at 765 nm using a Shimadzu UV‐1800 Spectrophotometer (Japan). Per 100 mg of extract, TPC was measured in mg gallic acid equivalents (mg GAE/100 mg). Measurement of TPC was done as mg GAE per 100 mg extract.

#### 2.3.2 Antioxidant Activity

The DPPH, FRAP, and ABTS assays were conducted on extracts of *H. rosa sinensis* flowers to determine their antioxidant potential using different solvents. DPPH was determined by following the method of Agunbiade et al. (2022), FRAP by Thaipong et al. (2006), and ABTS by the method of Bendaali et al. (2024), with minor adjustments. The DPPH absorbance was measured at 517 nm using a UV–Vis spectrophotometer (Velab 5100 UV) after incubating the mixture of 400 μL extract, 600 μL DPPH solution, and 200 μL distilled water. FRAP activity was measured by taking the absorbance at 593 nm after incubating the mixture of FRAP reagent and flower extract for 10 min. The FRAP unit was expressed as μmol of Trolox equivalent per 100 mL sample. ABTS antioxidant activity was determined by measuring the absorbance at 734 nm after incubating the mixture of 3 mL ABTS working solution and 30 μL flower extract in the dark for 16 h at 26°C. The DPPH and ABTS antioxidant activities were measured as % inhibition.

### 2.4 Gas Chromatography–Mass Spectrometry (GC–MS)

To identify the phytochemical constituents of *Hibiscus rosa‐sinensis* leaves, methanolic extract was characterized using gas chromatography and mass spectrometry model number (Agilent 5977B GC/MSD) using a thermal desorption system. The column size was 30 m × 0.25 mm × 0.25 μm. The starting temperature of the column was 100°C, which was steadily raised at the rate of 5°C per minute up to 280°C as this temperature was sustained for 3 min. This temperature was again raised at 15°C per min up to 280°C with a holding time of 35 min. The mass spectrometer ion source was kept at a temperature of 230°C with a 270°C interface temperature. Full scan mode detection was conducted from *m/z* 40 to 650. Determining constituents was done by comparing the mass spectra of obtained peaks with the NIST 20 (National Institute of Standards and Technology) and RTLPEST 3 libraries.

### 2.5 Fourier Transform Infrared Spectroscopy (FTIR) Scanning

Functional groups spectra of *Hibiscus rosa‐sinensis* flower were examined by Bruker Alpha FTIR spectrometer with Platinum crystal ATR, with spectral range from 400 to 4000 cm^−1^ and at resolution of 8 cm^−1^.

### 2.6 Lethal Dose Determination by Brine Shrimp Lethality Test

The shrimp lethality test depends on the ability to kill lab‐cultured Artemia nauplii/larvae brine shrimp. Larvae of the brine shrimp were mixed with previously prepared extract at the concentrations 1, 10, 100, 1000, 2000, and 5000 ppm each in a seawater solution of 10 mL with DMSO of 1% (v/v). Ten larvae were used for every test with replicates, and those that survived were counted after 24 h. Distilled water was used for the blank control. A lethal dose with a mortality of 50% after exposure of 24 h was measured using the probit method by Waghulde et al. (2019).

The current study determined that the lethality and extract concentration were directly proportional to the extent of lethality. All shrimps were alive after 24 h of observation in the control solution. Maximum mortality was observed at 5000 ppm, while the least was at 1 ppm concentration. It was observed that at higher concentrations of treatment extracts, the shrimps started dying only after 8 h, and after 24 h, all the shrimps died.

$$\text{Mortality percentage}=\left(\text{dead nauplii number}/\text{initial live nauplii number}\right)\times 100$$

#### 2.6.1 Test Solution Preparation

The methanol extract that was obtained after concentration was mixed in distilled water. The mixture was stirred thoroughly with a magnetic stirrer for 4–5 min. After every oral gavage administration, the solution was kept in the refrigerator. The following formula was used to select the solution volume

$$V=D^{*}P/C$$

where *V*, solution volume in mL; *D*, dose in mg/kg; *P*, animal weight in kg; *C*, solution concentration in mg/mL.

### 2.7 Animal Acclimatization

Rats (Sprague–Dawley) aged 7–8 weeks, weighing about 120 g ± 10 g, were obtained from the University of Veterinary and Animal Sciences, Lahore. In the animal room, rats were initially kept in 48‐h quarantine. After quarantine completion, they were shifted to the experimental room, where rats underwent acclimatization for 5 days.

#### 2.7.1 Animals Randomization and Husbandry

Rats were checked for thorough clinical assessment followed by weight on the digital weighing balance. Subsequently, 25 rats were randomly allotted to study groups based on their body weight. All the groups containing five animals were placed in well‐ventilated cages (5 rats per cage) with dimensions of 40 cm × 40 cm × 76 cm, with cob corn used as the bedding material. The temperature of the animal room was maintained at 23°C ± 2°C with relative humidity ranging from 45% to 60% and 12 light–dark cycles for a five‐day acclimatization period. Standard pellets were fed to animals during the study duration ad libitum with free access to reverse osmosis purified fresh water in polypropylene bottles.

#### 2.7.2 Ethics Declaration

The conformity of the study concerning the practices of animal husbandry and experimental procedures was set as per the standards of and it was in compliance with the guidelines of ARRIVE protocols (Du Sert et al. 2020). Approval of the study was taken from the Ethical Committee of the Faculty of Food Science and Nutrition, Bahauddin Zakariya University ethical approval board (Protocol Number: REC‐014), before the start of the study.

### 2.8 Sub‐Acute Toxicity

This study was designed as per the guidelines 407 for 28‐day oral toxicity for chemical analysis according to the Organization for Economic Cooperation and Development (OECD) in rodents OECD/OCED (1992). For the sub‐acute study, healthy rats were allocated to five groups, each group containing five. Groups that received the powder of flower in their diet were the 2nd and 3rd which were subjected to doses of 500 and 1000 mg/kg/day, while groups that received the extract were subjected to an oral gavage dose of 100 and 200 mg/kg/day. Toxicity signs were observed during the whole dosing period and the animals' body weights were measured for 28 days on a weekly basis. At the culmination of the study, all remaining animals were euthanized with ketamine and xylazine at concentrations of 85 and 9.1 mg/kg, respectively for blood collection using the retro‐orbital method.

### 2.9 Body Weights and Food Consumption

Weight of animals was observed on 7th, 14th, 21st and 28th day. Food intake of the animals in different groups was observed every week. Per capita water and food consumption for each week was taken by deploying the following formula:

$$\begin{array}{c} \mathrm{Per}\;\text{week water and feed consumption}:\\ \text{food given}\;\left(g\right)–\text{leftover food}\;\left(g\right)/\text{Animals numbers}\;\mathrm{per}\;\text{group} \end{array}$$

### 2.10 Oxidative Stress Markers in the Liver

#### 2.10.1 Preparation of Tissue Homogenates

Tissue of liver weighing 0.25 g of all groups was homogenized in PBS solution of 1 mL of 50 mM with EDTA 0.1 M pH 7.4, with centrifugation at 4°C for 20 min at 12,000×*g*. Supernatant was removed to determine biochemical markers. All tissue homogenates' protein concentration was measured by using the Bradford (1976) method with bovine serum albumin used as standard.

#### 2.10.2 Malondialdehyde Determination

The MDA concentration in 10% liver homogenates (prepared in NaCl 0.9%) was determined as thiobarbituric acid reactive substances as per the method described by Buege and Aust (1978).

#### 2.10.3 Glutathione (GSH)

Reduced glutathione was determined by having an assay mixture that consisted of liver homogenate of 62.5 μL, 0.2 M phosphate buffer 187.5 μL with pH 8.2, and 0.01 M DTNB of 12.5 μL (dithiobis‐nitrobenzoic acid). Afterward, absolute methanol of 987.5 μL was put in and placed inside a laboratory mixer for 15 min at 240 rpm, followed by centrifugation for 15 min at 3000 rpm (1260 x g) at room temperature. The yellow color was developed with a measurement of absorbance done at 412 nm. Units of the liver tissue were demonstrated as mM GSH/g tissue (Sedlak and Lindsay 1968).

#### 2.10.4 Catalase

Catalase activity was measured by following the decomposition in initial hydrogen peroxide concentration. The supernatant of 1 μL, which was removed from the liver homogenates of rats, was diluted in 0.1 M PBS with pH 7. A hydrogen peroxide concentration of 2 mM in a final volume of 1 mL was obtained by adding 1 M hydrogen peroxide to achieve a final volume in the cuvette. The change in the absorbance was measured at 240 nm for a time of two minutes with an interval of 30 s, and CAT activity was measured using a molar coefficient (ɛ = 43.6 Mcm^−1^) with enzyme activity expressed as U/mg protein (Aebi 1984).

#### 2.10.5 Superoxide Dismutase (SOD)

The SOD activity was determined by measuring its capacity to decrease nitrotetrazolium chloride photochemical reduction under controlled conditions. Superoxide anions produced via riboflavin‐mediated illumination react with SOD thus decreasing formazan product formation. Liver tissue homogenate of 10 μL in the assay mixture, with 0.067 M PBS of 641 μL at pH 7.0, 0.1 M EDTA of 40 μL, 1.5 mM blue nitrotetrazolium chloride of 20 μL and 0.1 mM riboflavin of 9 μL. The mixture was mixed gently followed by illumination by a 40‐watt lamp source at a 15 cm distance for 15 min and the measurement was done immediately at 560 nm by spectrophotometer. The blank sample with all the components except the liver tissue sample was not illuminated. One SOD unit was the amount of enzyme which is required for inhibition of 50% of NBT reduction rate under conditions of (1 min at 25°C), and it is expressed in U/mg protein (Bouhalit and Kechrid 2018).

$$\begin{array}{c} \text{Inhibition}\;\left(\%\right)=\\ \left(\left(A560\;\text{Absorbance of control}–\text{Sample}\;A560\right)/\text{Control}\;A560\right)\times 100 \end{array}$$

### 2.11 Hematology and Serum Biochemistry

Blood was collected in vials coated with EDTA as an anticoagulant. After mixing, blood was aspirated in the Cobas 6000 Auto Analyzer to quantify the following parameters: White Blood Cells (WBC) count and differential cells. Serum biochemistry analysis was done by collecting the rat's blood into gel vials without anticoagulants. Serum and plasma were obtained by centrifuging the clotted blood for 10 min at 3500 rpm (1710 x g) using a centrifuge (HERMLE Labortechnik GmbH, D‐78564, Z 326 K, Wehingen, Germany). The serum was engaged to determine electrolytes, i.e., Sodium (Na), Chloride (Cl), and Potassium (K), while plasma was used for the determination of the following parameters: Alanine Aminotransferase (ALAT), Aspartate aminotransferase (ASAT), Alkaline Phosphate (ALP), Bilirubin, Urea, Uric acid, and Creatinine, using the Lisa 300 Hycel automaton.

### 2.12 Lipid and Protein Profile

Lipid profile, which includes high‐density lipoprotein (HDL) was measured by the method described by Albers et al. (1978) and serum total cholesterol (TC) by the method of Allain et al. (1974). VLDL was determined by dividing triglycerides by five using the method of Warnick et al. (1990), while LDL was determined by using the Friedewald formula of Friedewald et al. (1972) and triglycerides (TG) by the method described by Bucolo and David (1973). Total protein determination was done by the method of Lowry of Sarkar et al. (2020).

### 2.13 Histopathology Evaluation

Animals were subjected to dissection post retro‐orbital blood collection for organ collection. Kidneys and liver were removed with no attachment of any other tissue and weighed to determine the relative weight. Selected weighed organs were moved in a 10% solution of Neutral Buffered Formalin for histological analysis. Fixed organ tissues were embedded in the paraffin at the tissue embedding station. Afterward, a microtome was used to slice the sections of tissues to a thickness of 3–5 μm. These sliced sections underwent staining with hematoxylin–eosin. These sections, after staining, were subjected to observation under a microscope for any alterations (Nagy and Ewais 2014).

### 2.14 Statistical Analysis

Data from the current study were presented as means ± SEM (four replicates). Statistical significance with a 5% significance level (*p* < 0.05) was conducted using the ANOVA test. Graphs were prepared on GraphPad Prism 8.0.1.

## 3 Results

### 3.1 *Hibiscus rosa‐sinensis* Extracts Total Phenolic Content and Antioxidant Activity

**FIGURE 1:**
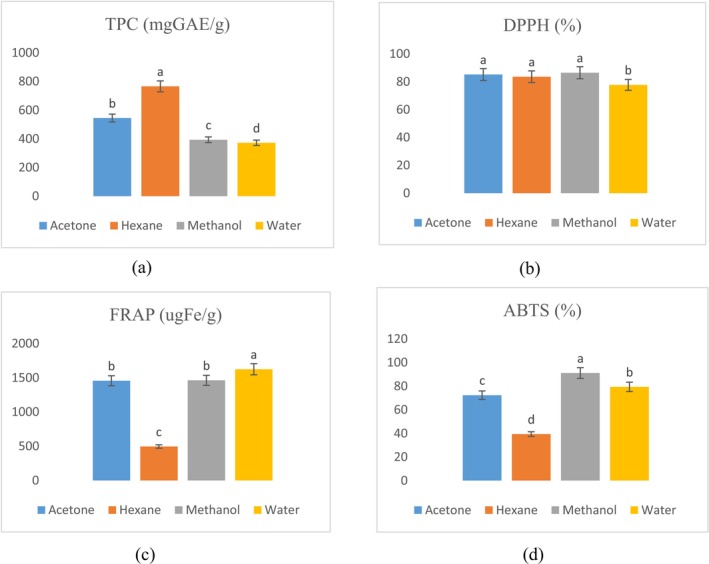
*Hibiscus rosa‐sinensis* flowers (a) TPC (b) DPPH (c) FRAP and (d) ABTS, activity in different solvents.

Total phenolic compounds and antioxidant potential of *Hibiscus rosa‐sinensis* in various solvents through TPC, DPPH, FRAP, and ABTS are depicted in Figure 1a–d. The results regarding TPC revealed the highest value in hexane and the lowest in distilled water (DW), with values of 765.72 and 372.27 mgGAE/g. The DPPH assay showed that the highest result was observed in methanol with the value of 86.459%, followed by 85.182% and 83.591% in acetone and hexane, respectively. The FRAP assay showed 1623.3, 1461.9, and 1455.5 μgFe/g in distilled water, methanol, and acetone solvents, respectively. The highest value, 91.12% in ABTS, was explicated by the methanolic extract, 79.394% by distilled water, and 72.325% by acetone solvent.

### 3.2 Phytochemical Compounds Identification of *Hibiscus rosa‐sinensis* by Gas Chromatography–Mass Spectrometry (GC–MS)

**FIGURE 2:**
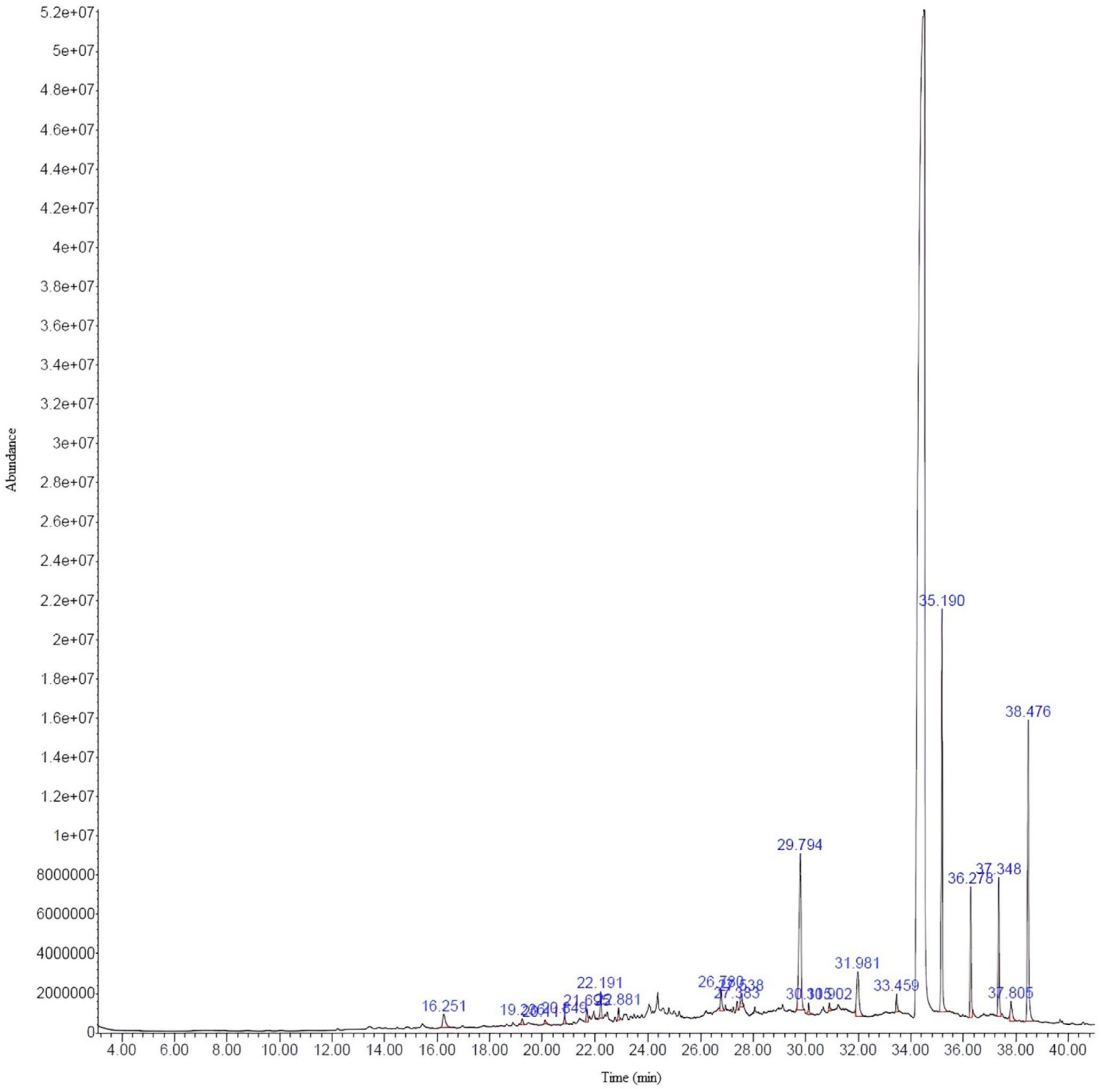
GC–MS chromatogram of *Hibiscus rosa‐sinensis* flower methanol extract bioactive compounds.

Phytochemical compounds of *Hibiscus rosa‐sinensis* by gas chromatographic profile highlighted peak number and the retention time along with compound name present in methanolic extract of the plant (Table 1). Every compound is presented with a dominant percentage. The chromatogram of each peak of the detected compound is shown in Figure 2. GC–MS analysis of plant methanolic extract chromatogram compounds concerning the peak is Pentadecanal as the first peak followed by cis‐11‐Hexadecenal. Main compounds of the plant methanolic extract concerning the area percentage are 1,2‐Cyclohexanedicarboxylic acid, bis(2‐ethylhexyl) ester, 2‐[3‐Chlorophenoxy] propionamide (25.29 min) from two different libraries, 1,2‐Cyclohexanedicarboxylic acid, bis(2‐ethylhexyl) ester and 2‐[3‐Chlorophenoxy] propionamide (17.77 min). In Table 1, other main compounds that were identified are 1,4‐Benzenedicarboxylic acid, bis(2‐ethylhexyl) ester, 2‐Butenedioic acid, bis(2‐ethylhexyl) ester and Malathion‐o‐analog (7.19 min), 2‐Butenedioic acid (E)‐, bis(2‐ethylhexyl) ester and 4‐Chloroaniline (5.82 min).

**TABLE 1:** Compounds identified in plant methanolic extract of *Hibiscus rosa‐sinensis* by GC.

Peak no.	R. T (min)	Name	Area (%)
1	16.251	Pentadecanal	1.89
2	19.236	cis‐11‐Hexadecenal	0.53
3	20.111	Hexadecanal	0.32
4	20.849	2‐Pentadecanone, 6,10,14‐trimethyl—	0.56
5	21.695	9‐Tetradecen‐1‐ol, acetate, (Z)—	1.14
6	22.191	Cyclopropaneoctanal, 2‐octyl—	1.78
7	22.881	Oxirane, heptadecyl—	0.81
8	26.780	2 (3H)‐Furanone, 5‐dodecyldihydro—	1.69
9	27.383	2H‐Pyran‐2‐one, tetrahydro‐6‐undecyl, Tributyl phosphate	0.55
10	27.538	9, 12‐Octadecadienoic acid (Z,Z)—	1.12
11	29.794	1,2‐Cyclohexanedicarboxylic acid, bis (2‐ethylhexyl) ester, 2‐[3‐Chlorophenoxy]propionamide	17.77
12	30.115	(Z)‐9‐octadecen‐4‐olide	0.65
13	30.902	Benzo[f]quinolone	0.50
14	31.981	2‐Butenedioic acid (E)‐, bis (2‐ethylhexyl) ester 4‐Chloroaniline	5.82
15	33.459	Bis(2‐ethylhexyl) phthalate	1.19
16	35.190	1,2‐Cyclohexanedicarboxylic acid, bis (2‐ethylhexyl) ester 2‐[3‐Chlorophenoxy]propionamide	25.29
17	36.278	2‐Butenedioic acid (E)‐, bis (2‐ethylhexyl) ester Malathion‐o‐analog	7.19
18	37.348	1,4‐Benzenedicarboxylic acid, bis (2‐ethylhexyl) ester	8.02

### 3.3 FTIR Analysis of *Hibiscus rosa‐sinensis* Flower Powder and Methanolic Extract

**FIGURE 3:**
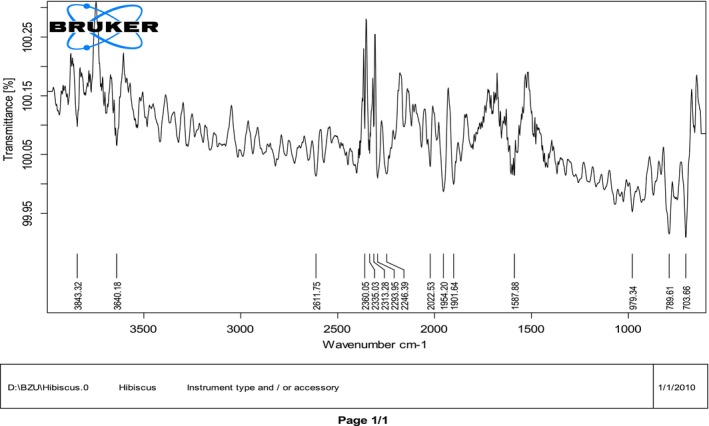
FTIR scanning for *Hibiscus rosa‐sinensis* flower powder.

**FIGURE 4:**
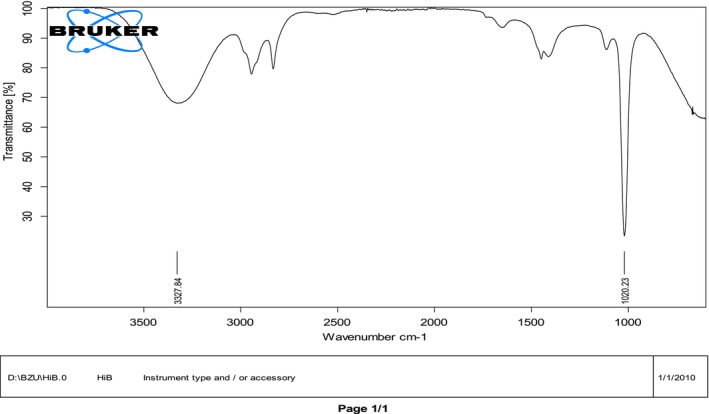
FTIR scanning for *Hibiscus rosa‐sinensis* flower methanol extract.

FTIR analysis of *Hibiscus rosa‐sinensis* flower powder and methanol extract in Figures 3, 4 indicated the presence of multiple functional groups linked with bioactive compounds. The peaks at 3843.32 and 3640.18 cm^−1^ correspond to O–H stretching, typically found in hydroxyl‐containing molecules such as phenolic compounds, flavonoids, and water. A peak at 2611.75 cm^−1^ showed the presence of carboxylic acid (–COOH) groups, commonly found in organic acids. The area between 2390.05 and 2249.50 cm^−1^ exhibited characteristic carbon dioxide or nitriles (C≡N) stretching, which could be linked to alkaloid structures or other nitrogen‐containing phytochemicals. Additionally, peaks at 2022.53 and 1901.64 cm^−1^ indicated C=O stretching, the presence of conjugated ketones or aldehydes, typically associated with flavonoids. A strong absorption at 1677.88 cm^−1^ attributed to carbonyl (C=O) stretching indicated polyphenolic compounds, which contribute to the antioxidant and therapeutic properties of *Hibiscus rosa‐sinensis* . The spectral bands at 979.34, 789.61, and 703.66 cm^−1^ correspond to C–H bending vibrations in the aromatic rings, confirming benzene derivatives' presence, likely flavonoids or tannins. Overall, these spectral characteristics highlighted the fullness of *Hibiscus rosa‐sinensis* in phytoconstituents such as flavonoids, polyphenols, and organic acids, all of which play important roles in pharmacological and medicinal activities.

### 3.4 Feed Consumption

**FIGURE 5:**
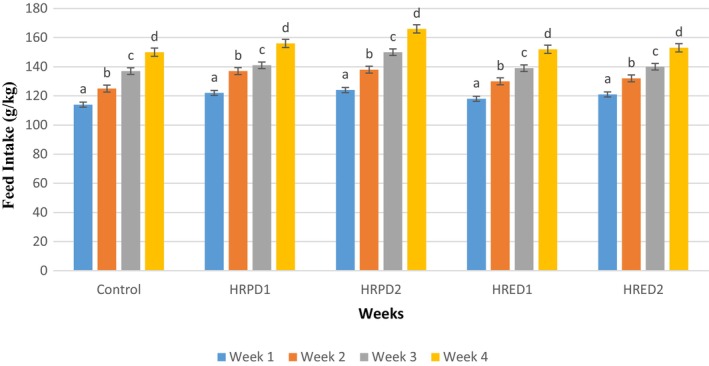
Feed intake of the control group with respect to treatment groups during 4‐week study duration of *Hibiscus rosa‐sinensis* flower safety.

The feed intake of different groups fed on *Hibiscus rosa‐sinensis* flowers' powder and extract for 4 weeks is depicted in Figure 5. The feed intake showed that the maximum feed was consumed by the placebo and *Hibiscus* powder 1000 mg/kg group, compared with other supplemented groups. The extract groups consumed less feed than powder‐supplemented groups, and minimum feed intake was observed in the extract 2 group supplemented with 200 mg/kg BW extract. However, the feed intake gradually improved in study duration between weeks, and the highest consumption was noticed in the 4th week of the study in all supplemented groups.

### 3.5 Body Weight

The effect of *Hibiscus rosa‐sinensis* flower extract and powder on the rodent body and organ weight showed significant (*p* <> 0.05) variations in body weight. In contrast, non‐significant changes in organ weight and results are displayed in Table 2. The body weight improved to 218.33 ± 9.86 g and 229.89 ± 9.73 g in *Hibiscus rosa‐sinensis* powder diet 1 and *Hibiscus rosa‐sinensis* powder diet 2 powder groups supplemented with 500 and 1000 mg/kg BW powder, respectively. The liver and heart weights were increased in extract groups, compared with placebo and powder groups. The weights augmented to 8.02 ± 0.21, 8.09 ± 0.14, 0.42 ± 0.02, and 0.41 ± 0.03 g in both extract groups. Both kidneys exhibited minor weight fluctuations, and the highest value was noticed in *Hibiscus rosa‐sinensis* extract diet 1 for the left and right kidneys. The highest values were 0.47 ± 0.02 and 0.57 ± 0.02 g, shown by *Hibiscus rosa‐sinensis* extract diet 2, respectively. The spleen and lung weights enhanced to 0.52 ± 0.02 g and 1.55 ± 0.11 g in extract groups from 0.46 ± 0.02 g and 1.55 ± 0.04 g, respectively.

**TABLE 2:** Effect of *Hibiscus rosa‐sinensis* flower extract and powder on the rodent body and organ weight.

Parameters (g)	Treatments					*p*
	Control	HRPD1	HRPD2	HRED1	HRED2	
Body weight	208.02 ± 5.01^e^	218.33 ± 9.86^d^	229.89 ± 9.73^b^	234.79 ± 4.85^a^	226.19 ± 3.33^c^	0.007*
Liver	7.80 ± 0.21^a^	7.88 ± 0.47^a^	7.93 ± 0.29^a^	8.02 ± 0.21^a^	8.09 ± 0.14^a^	0.751^ns^
Heart	0.37 ± 0.02^a^	0.39 ± 0.02^a^	0.40 ± 0.02^a^	0.42 ± 0.02^a^	0.41 ± 0.03^a^	0.121^ns^
Kidney L	0.42 ± 0.01^a^	0.44 ± 0.03^a^	0.46 ± 0.02^a^	0.47 ± 0.02^a^	0.45 ± 0.02^a^	0.100^ns^
Kidney R	0.52 ± 0.01^a^	0.55 ± 0.02^a^	0.55 ± 0.01^a^	0.56 ± 0.01^a^	0.57 ± 0.02^a^	0.17ns
Spleen	0.46 ± 0.02^a^	0.48 ± 0.02^a^	0.51 ± 0.01^a^	0.52 ± 0.02^a^	0.50 ± 0.02^a^	0.27^ns^
Lungs	1.55 ± 0.04^a^	1.54 ± 0.02^a^	1.53 ± 0.07^a^	1.52 ± 0.08^a^	1.55 ± 0.11^a^	0.970^ns^

*Note:* *P* < 0.05; *, significant; ns, non‐significant; Control, negative control group; HRPD1, *Hibiscus rosa‐sinensis* flower powder dose 1 (500 mg); HRPD2, *Hibiscus rosa‐sinensis* flower powder dose 2 (1000 mg); HRED1, *Hibiscus rosa‐sinensis* flower extract dose 1 (100 mg); HRED2, *Hibiscus rosa‐sinensis* flower extract dose 2 (200 mg). Different superscript letters in a row indicate statistical differences between treatments at p < 0.05.

### 3.6 Renal Function Test

Renal function test (RFT) comprising creatinine, urea, uric acid, sodium (Na), and potassium (K) shows statistically significant results (*p* < 0.05), presented in Table 3. The creatinine and urea levels increased in supplemented groups, compared with placebo. The increased creatinine and urea levels are 0.59 ± 0.01 and 28.01 ± 1.01 mg/dL in placebo, whilst amplified to 0.61 ± 0.02, 0.65 ± 0.04, 1.75 ± 1.30, and 35.28 ± 0.51 mg/dL in HRPD1 and HRPD2 respectively, supplemented with 500 and 1000 mg/kg BW flower powder. Moreover, the creatinine increased in extract groups compared with powder groups. The uric acid amplified non‐significantly in supplemented groups, and the highest values were observed in both extract groups with readings of (1.95 ± 0.09 and 1.98 ± 0.11 mg/dL). The Na depicted non‐significant changes, as the value declined to 131.45 ± 5.75 and 32.89 ± 5.71 mEq/L in both powder groups, compared with placebo 136.05 ± 6.81 mEq/L. The K values augmented in supplemented groups compared with placebo, showing significant (*p* < 0.05) variations. The highest K values were noticed in HRPD2 1000 mg/kg BW powder and HRED2 200 mg/kg BW extract groups, with values of (34.45 ± 1.83 and 35.77 ± 1.56 mmol/L), respectively.

**TABLE 3:** Effect of *Hibiscus rosa‐sinensis* flower extract and powder on the renal function test.

RFTs	Treatments					*p*
	Control	HRPD1	HRPD2	HRED1	HRED2	
Creatinine (mg/dL)	0.59 ± 0.01^a^	0.61 ± 0.02^a^	0.65 ± 0.04^a^	0.69 ± 0.02^a^	0.71 ± 0.03^a^	0.16^ns^
Urea (mg/dL)	28.01 ± 1.01^d^	31.75 ± 1.30^c^	35.28 ± 0.51^a^	33.50 ± 2.44^b^	35.81 ± 1.04^a^	0.03*
Uric Acid (mg/dL)	1.87 ± 0.09^a^	1.88 ± 0.08^a^	1.94 ± 0.08^a^	1.95 ± 0.09^a^	1.98 ± 0.11^a^	0.521^ns^
Na (mEq/L)	136.05 ± 6.81^a^	131.45 ± 5.75^b^	132.89 ± 5.71^b^	135.21 ± 7.12^a^	136.33 ± 8.44^a^	0.878^ns^
K (mmol/L)	29.55 ± 0.56^d^	32.48 ± 1.50^c^	34.45 ± 1.83^a^	30.61 ± 1.49^d^	35.77 ± 1.56^a^	0.04*

*Note:* *P* < 0.05; *, significant; ns, non‐significant; Control, negative control group; HRPD1, *Hibiscus rosa‐sinensis* flower powder dose 1 (500 mg); HRPD2, *Hibiscus rosa‐sinensis* flower powder dose 2 (1000 mg); HRED1, *Hibiscus rosa‐sinensis* flower extract dose 1 (100 mg); HRED2, *Hibiscus rosa‐sinensis* flower extract dose 2 (200 mg). Different superscript letters in a row indicate statistical differences between treatments at p < 0.05.

### 3.7 Liver Function Test

**FIGURE 6:**
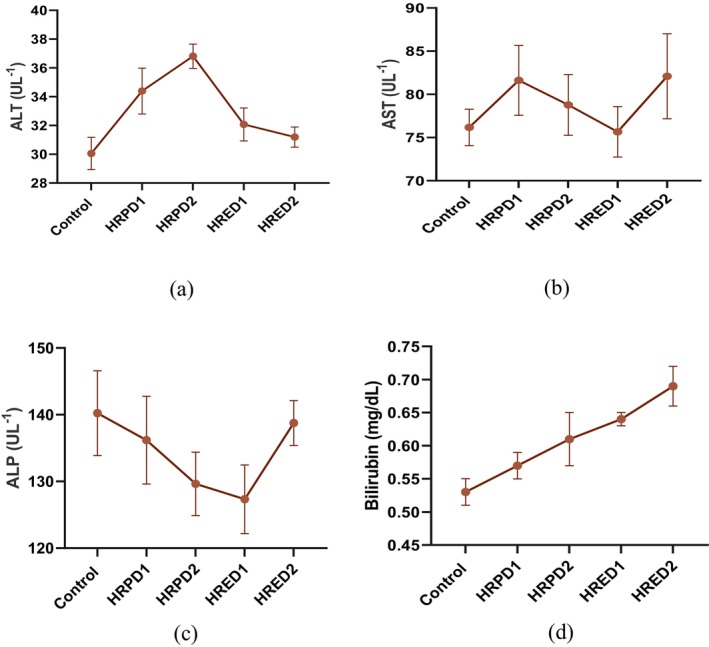
Liver function test parameters, that is, ALT, AST, ALP and Bilirubin, Control, negative control group; HRPD1, *Hibiscus rosa‐sinensis* flower powder dose 1 (500 mg); HRPD2, *Hibiscus rosa‐sinensis* flower powder dose 2 (1000 mg); HRED1, *Hibiscus rosa‐sinensis* flower extract dose 1 (100 mg); HRED2, *Hibiscus rosa‐sinensis* flower extract dose 2 (200 mg).

The comparative analysis of different doses of *Hibiscus rosa‐sinensis* flower extract and powder on the liver function test (LFT) revealed significant (*p* < 0.05) variations in ALT and bilirubin, while non‐substantial changes in AST and ALP, and results are demonstrated in Figure 6a–d. The AST values augmented in both powder groups and the values are 81.62 ± 4.04 and 78.77 ± 3.52 UL^−1^, compared to placebo 76.17 ± 2.11 UL^−1^. The ALT values increased in all supplemented groups, and the highest value was recorded in HRPD2, followed by HRPD1 group. The HRPD1 and HRPD2 revealed a decline in ALP results, and the values are (136.18 ± 6.57 and 129.64 ± 4.74 UL^−1^), compared with placebo 140.23 ± 6.35 UL^−1^. The lowest ALP value was indicated by the 100 mg/kg BW extract group. The bilirubin levels increased in both extract groups, compared with powder groups and placebo.

### 3.8 Protein Content

The Table 4 presenting the serum protein profile, comprising total protein, albumin, globulin, and A/G ratio, showed non‐significant results. The serum total protein was reduced in all supplemented groups, except the HRPD2 group, compared to the placebo. The placebo indicated 7.93 ± 0.33 g/dL, while values dropped to 7.59 ± 0.21 and 7.62 ± 0.42 g/dL in HRPD1 500 mg/kg BW powder and HRED2 200 mg/kg BW extract groups, respectively. The albumin increased to 4.36 ± 0.04 g/dL in the HRPD2 group, compared to placebo 4.25 ± 0.14 g/dL and other supplemented groups. The globulin declined in all supplemented groups with the values of (3.45 ± 0.14, 3.65 ± 0.24, 3.63 ± 0.14, 3.50 ± 0.19 g/dL) in HRPD1, HRPD2, HRED1, HRED2, respectively. The A/G ratio improved to 1.20 ± 0.02, 1.19 ± 0.03, 1.16 ± 0.04, and 1.17 ± 0.03 in all supplemented groups.

**TABLE 4:** Effect of *Hibiscus rosa‐sinensis* flower extract and powder on the protein content.

Proteins	Treatments					*p*
	Control	HRPD1	HRPD2	HRED1	HRED2	
Total Protein (g/dL)	7.93 ± 0.33^a^	7.59 ± 0.21^a^	8.01 ± 0.01^a^	7.84 ± 0.24^a^	7.62 ± 0.42^a^	0.318^ns^
Albumin (g/dL)	4.25 ± 0.14^a^	4.15 ± 0.19^a^	4.36 ± 0.04^a^	4.21 ± 0.17^a^	4.10 ± 0.15^a^	0.32^ns^
Globulin (g/dL)	3.68 ± 0.22^a^	3.45 ± 0.14^a^	3.65 ± 0.24^a^	3.63 ± 0.14^a^	3.50 ± 0.19^a^	0.53^ns^
A/G Ratio	1.15 ± 0.06^a^	1.20 ± 0.02^a^	1.19 ± 0.03^a^	1.16 ± 0.04^a^	1.17 ± 0.03^a^	0.53^ns^

*Note:* *P* < 0.05; ns, non‐significant; Control, negative control group; HRPD1, *Hibiscus rosa‐sinensis* flower powder dose 1 (500 mg); HRPD2, *Hibiscus rosa‐sinensis* flower powder dose 2 (1000 mg); HRED1, *Hibiscus rosa‐sinensis* flower extract dose 1 (100 mg); HRED2, *Hibiscus rosa‐sinensis* flower extract dose 2 (200 mg). Different superscript letters in a row indicate statistical differences between treatments at p < 0.05.

### 3.9 Lipid Profile

The comparative impact of different doses of *Hibiscus rosa‐sinensis* flower powder and extract showed significant (*p* < 0.05) variations in lipid profile parameters (glucose, triglycerides, and VLDL), whilst non‐significant changes in total cholesterol, LDL, HDL, and AIP values. The glucose was reduced to 78.33 ± 4.62 and 74.12 ± 3.10 mg/dL in HRPD1 and HRPD2 groups, supplemented with 500 and 1000 mg/kg BW powder, respectively. The triglycerides decreased from 75.87 ± 2.65 to 61.39 ± 2.15 mmol/L. The total cholesterol dropped to 89.34 ± 3.42, 87.22 ± 3.32, and 85.92 ± 4.76 mmol/L in HRPD1, and HRED2 groups, respectively. The VLDL reduced significantly in extract groups with the values of (12.70 ± 0.45 and 12.27 ± 0.49 mmol/L). At the same time, LDL exhibited non‐significant changes, and the highest decline was noticed in both extract groups with the values of (39.71 ± 1.66 and 39.03 ± 1.03 mmol/L). The HDL depicted the same unsubstantial trend, reducing values in extract groups. The AIP value augmented in the HRPD1 group with the value of (0.33 ± 0.03), whereas it decreased to 0.30 ± 0.02 and 0.27 ± 0.02 in HRPD2 1000 mg/kg BW powder and HRED2 200 mg/kg BW extract groups, respectively (Table 5).

**TABLE 5:** Effect of *Hibiscus rosa‐sinensis* flower extract and powder on the lipid profile.

Lipid Profile	Treatments					*p*
	Control	HRPD1	HRPD2	HRED1	HRED2	
Glucose (mg/dL)	84.64 ± 4.41^a^	78.33 ± 4.62^b^	74.12 ± 3.10^c^	72.04 ± 3.28^d^	69.94 ± 0.25^e^	0.0027*
TG (mmol/L)	75.87 ± 2.65^a^	69.53 ± 0.67^b^	64.92 ± 1.58^c^	63.51 ± 1.98^c^	61.39 ± 2.15^d^	0.03*
TC (mmol/L)	93.48 ± 3.17^a^	90.66 ± 5.25^b^	89.34 ± 3.42^b^	87.22 ± 3.32^c^	85.92 ± 4.76^d^	0.02*
VLDL (mmol/L)	15.17 ± 0.60^a^	13.91 ± 0.22^b^	12.98 ± 0.44^c^	12.70 ± 0.45^c^	12.27 ± 0.49^c^	0.04*
LDL (mmol/L)	41.87 ± 1.26^a^	41.14 ± 0.83^a^	40.57 ± 3.30^a^	39.71 ± 1.66^a^	39.03 ± 1.03^a^	0.401^ns^
HDL (mmol/L)	36.44 ± 1.48^a^	35.61 ± 1.53^a^	35.77 ± 1.50^a^	34.81 ± 0.66^a^	34.60 ± 0.60^a^	0.407^ns^
AIP	0.30 ± 0.01^a^	0.33 ± 0.03^a^	0.30 ± 0.02^a^	0.31 ± 0.03^a^	0.27 ± 0.02^a^	0.351^ns^

*Note:* *P* < 0.05; *, significant; ns, non‐significant; Control, negative control group; HRPD1, *Hibiscus rosa‐sinensis* flower powder dose 1 (500 mg); HRPD2, *Hibiscus rosa‐sinensis* flower powder dose 2 (1000 mg); HRED1, *Hibiscus rosa‐sinensis* flower extract dose 1 (100 mg); HRED2, *Hibiscus rosa‐sinensis* flower extract dose 2 (200 mg). Different superscript letters in a row indicate statistical differences between treatments at p < 0.05.

### 3.10 Oxidative Stress Biomarkers of Liver Tissue Homogenate

**FIGURE 7:**
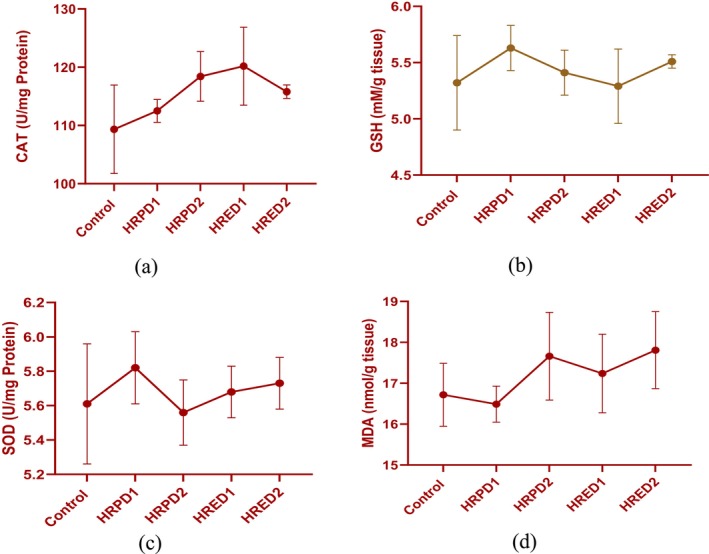
*Hibiscus rosa‐sinensis flower* extract and powder effect on oxidative stress biomarkers, Control, negative control group; HRPD1, *Hibiscus rosa‐sinensis* flower powder dose 1 (500 mg); HRPD2, *Hibiscus rosa‐sinensis* flower powder dose 2 (1000 mg); HRED1, *Hibiscus rosa‐sinensis* flower extract dose 1 (100 mg); HRED2, *Hibiscus rosa‐sinensis* flower extract dose 2 (200 mg).

The oxidative stress markers of liver tissue homogenate showed non‐significant variations in values, and results are displayed in Figure 7a–d. The CAT, GSH, and SOD slightly improved in supplemented groups compared with placebo. The CAT improved in HRED1 supplemented with 100 mg/kg BW extract. The GSH improved in HRPD1, and HRPD2, while it was reduced in HRED1. The SOD improved in all supplemented groups, and the highest improvement was shown by the HRPD1 and HRED2 groups. The MDA was reduced in the HRPD1 500 mg/kg BW powder group while it slightly increased in other supplemented groups.

### 3.11 Blood Indices (White Blood Cells and Differentials)

The results comprising white blood cell (WBC) and differential cell count showed non‐significant changes, and results are presented in Table 6. However, the values substantially changed in monocytes and basophils. The WBC slightly increased in supplemented groups, and HRED1 and HRED2 groups showed the highest results with the values of (8.86 ± 0.24 and 9.02 ± 0.34 × 10^3^/μL) respectively. The lymphocytes were reduced in powder groups with the values of (58.21% ± 1.72% and 57.70% ± 2.12%), compared with placebo 58.60% ± 3.03%. The neutrophils marginally amplified in both powder groups, supplemented with 500 mg/kg and 1000 mg/kg BW powder, while reduced in HRED2 200 mg/kg BW extract group, and the values are (38.56% ± 1.06%, 38.83% ± 1.18%, and 37.77% ± 0.48%), respectively. The monocytes augmented in powder and HRED1 groups compared with placebo; the observed values were 0.64% ± 0.03%, 0.71% ± 0.02%, and 0.74% ± 0.05%, respectively. The eosinophils decreased to 2.44% ± 0.07% in HRPD1, compared with placebo 2.48% ± 0.08%; however, they improved to 2.60% ± 0.09% in HRPD2 1000 mg/kg BW powder. The basophils non‐significantly increased in all supplemented groups, and the highest values of 0.153% ± 0.01% and 0.156% ± 0.01% were observed in both extract groups.

**TABLE 6:** Effect of *Hibiscus rosa‐sinensis* flower extract and powder on the white blood cells and differential cells.

Blood indices	Treatments					*p*
	Control	HRPD1	HRPD2	HRED1	HRED2	
WBC (×10^3^/μL)	8.12 ± 0.11^b^	8.36 ± 0.20^ab^	8.62 ± 0.34^ab^	8.86 ± 0.24^a^	9.02 ± 0.34^a^	0.11^ns^
Lymphocytes (%)	58.60 ± 3.03^a^	58.21 ± 1.72^a^	57.70 ± 2.12^a^	58.45 ± 1.90^a^	58.85 ± 3.67^a^	0.985^ns^
Neutrophils (%)	38.23 ± 2.08^a^	38.56 ± 1.06^a^	38.83 ± 1.18^a^	38.17 ± 2.49^a^	37.77 ± 0.48^a^	0.941^ns^
Monocytes (%)	0.58 ± 0.02^b^	0.64 ± 0.03^b^	0.71 ± 0.02^a^	0.74 ± 0.05^a^	0.57 ± 0.02^b^	0.79^ns^
Eosinophil (%)	2.48 ± 0.08^a^	2.44 ± 0.07^a^	2.60 ± 0.09^a^	2.49 ± 0.16^a^	2.64 ± 0.09^a^	0.126^ns^
(%)	0.096 ± 0.01^b^	0.146 ± 0.01^a^	0.150 ± 0.01^a^	0.153 ± 0.01^a^	0.156 ± 0.01^a^	0.15^ns^

*Note:* ns, non‐significant; Control, negative control group; HRPD1, *Hibiscus rosa‐sinensis* flower powder dose 1 (500 mg); HRPD2, *Hibiscus rosa‐sinensis* flower powder dose 2 (1000 mg); HRED1, *Hibiscus rosa‐sinensis* flower extract dose 1 (100 mg); HRED2, *Hibiscus rosa‐sinensis* flower extract dose 2 (200 mg). Different superscript letters in a row indicate statistical differences between treatments at p < 0.05.

### 3.12 Histology Analysis

**FIGURE 8:**
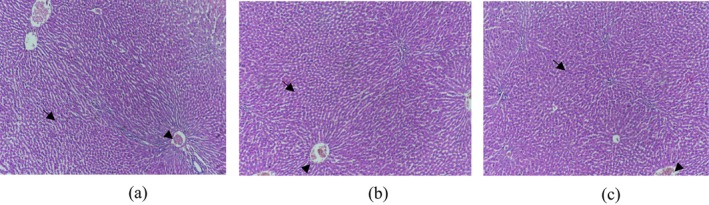
Rats' liver photomicrographs at ×200 magnification (a) control group (b) *Hibiscus rosa‐sinensis* flower powder 1000 mg/kg (c) *Hibiscus rosa‐sinensis* flower extract 200 mg/kg.

The liver samples collected from the experimental Sprague Dawley rats at the end of the sub‐acute toxicity study were fixed in 10% neutral buffered formalin for histological analysis using the paraffin sectioning technique and H&E staining procedure. In the hepatic parenchyma there were prominent round nuclei of hepatocytes along with the presence of sinusoidal spaces (star). Photomicrographs from the control and upper‐range dose (1000 and 200 mg/kg) groups, which offered experimental diet in powder and in methanol extract showed no significant morphological alterations in hepatic parenchyma when observed under the microscope (*p <* 0.05) (Figure 8a–c). The hepatocytes had prominent centrally placed round nuclei (arrow) and no prominent degeneration or inflammatory response except at some places where a slight congestive response (arrow head) was observed in the blood vessels. All the tissue sections were stained with H&E staining (×200).

## 4 Discussion

Medicinal plants and herbs are admired due to their phytochemistry and health benefits. The current research study investigates the phytochemistry and safety of *Hibiscus rosa‐sinensis* flower through GC–MS analysis, FTIR spectroscopy, and sub‐acute toxicity in rat modeling. The results regarding antioxidant activity showed that the methanolic extract exhibited the highest antioxidant potential in the DPPH and ABTS assays, while hexane and DW revealed the highest potential in the TPC and FRAP assays. Our findings are in accordance with Khan et al. (2014), who investigated the antioxidant potential of *H. sinensis* flowers and reported a high antioxidant potential (75.46% ± 4.67%) for the methanolic extract, compared with the ethanolic extract. Moreover, phytochemical profiling exhibited a diverse range of bioactive compounds in the methanolic extract of *Hibiscus rosa‐sinensis* , and 20 compounds were identified, and ethyl esters are the dominating compounds. However, other compounds with dominant percentage are 1,2‐Cyclohexanedicarboxylic acid, bis (2‐ethylhexyl) ester 2‐[3‐Chlorophenoxy] propionamide (25.29%), and 1,2‐Cyclohexanedicarboxylic acid, bis (2‐ethylhexyl) ester, 2‐[3‐Chlorophenoxy] propionamide (17.77%), respectively. In a study, the GC–MS analysis revealed that phthalates are the major compounds in the methanolic extract of *Hibiscus rosa‐sinensis* flowers (Yasmin 2023). Previously, another study of *Hibiscus* flower GC–MS analysis reported the presence of fifteen compounds, and ethyl esters were dominant compounds in the methanolic extract (Rassem et al. 2017). The presence of O–H, –COOH, C≡N, C=O, and C–H functional groups linked with different peaks in FTIR spectroscopy shows the existence of flavonoids, polyphenols, tannins, alkaloids, and organic acids in *Hibiscus rosa‐sinensis* flowers. Our findings of FTIR analysis are in accordance with the results of Abate and Belay (2022), as they conducted UV–Vis spectra and FTIR analysis and reported the presence of O–H, C–O, and C–H functional groups, thus evidencing the occurrence of phenolic compounds such as tannin and flavonoid in *Hibiscus rosa‐sinensis* flowers. The findings of Gomare and Mishra (2018) reported that the ethanolic extract of *Hibiscus rosa‐sinensis* is found to be rich in C=O, C–H, and C–N functional groups, while the methanolic extract is rich in N–H and S–S groups. Thus, based on these studies and our results, flavonoids, polyphenols, tannins, alkaloids, and organic acids are major bioactive compounds in *Hibiscus rosa‐sinensis* flowers.

The feed intake slightly improved in supplemented rats, although *Hibiscus rosa‐sinensis* has a tart, tangy, sour flavor and high fiber content. In addition, the flowers are high in protein and carbohydrate content, and protein has been reported to induce a thermogenic effect, leading to increased metabolism and, eventually, more feed consumption. Moreover, the body weight also slightly improved in rats, associated with protein and carbohydrate content. Therefore, the findings of this study endorse its application in weight management, and the studies on *Hibiscus* varieties proved their effectiveness in weight management. Concerning this, a randomized, double‐blind, placebo‐controlled trial proved that the extract of *Lippia citriodora* and *H. sabdariffa* controlled appetite sensation in overweight subjects (Serna et al. 2022). The weight management effects of *Hibiscus* varieties are attributed to their prebiotic properties, which are involved in the modulation of gut microbiota. In a study, phenolic extracts from *H. sabdariffa* were administered orally in obese rats for 42 days, and it was found that the extract effectively managed weight, improved glucose tolerance, insulin sensitivity, and regulated LDL/HDL ratio in obese mice (Diez‐Echave et al. 2020). The serum glucose and lipid profile were reduced in supplemented rats, and the 200 mg/kg extract group showed the highest reduction. The decreased serum lipid profile shows its antidiabetic and hypolipidemic potential. The hypoglycemic and lipid‐lowering effect is attributed to the nutritional composition and phytochemistry of *Hibiscus rosa‐sinensis*. The soluble fiber is linked with gel formation in the stomach, resulting in slow digestion and delayed release of glucose in the blood, eventually leading to low blood glucose levels and hypoglycemia. Moreover, fiber also regulates the protein expression of lipid metabolism and thus reduces circulating lipid levels via modulating PPAR‐γ and p‐SREBP‐1C expression (Jayachandran et al. 2023). Besides glucose alleviation, soluble dietary fiber (SDF) stimulates short‐chain fatty acids (SCFAs) production in the large intestine. These SCFAs activate AMPK in intestinal epithelial cells, skeletal muscle, and liver cells. The AMPK activation is another central mechanism responsible for hypoglycemic and hypolipidemic impact (Wu et al. 2021). The antidiabetic and hypocholesterolemic role of *Hibiscus rosa‐sinensis* was reported in various studies (Sanadheera et al. 2021). Besides *Hibiscus rosa‐sinensis* , other varieties of the genus *Hibiscus* are extensively documented with various therapeutic potential and safe outcomes. A study on *H. sabdariffa* powder reported its valuable impact on body weight and insulin resistance. The findings showed that the powder reduced obesity, decreased body weight, and improved insulin resistance (Amaya‐Cruz et al. 2019).

The serum protein profile, LFT, and RFT showed variations in supplemented groups, and values remained in the normal range. The ALT and bilirubin values marginally increased in the 1000 mg/kg powder and 200 mg/kg extract groups. Likewise, the serum creatinine, urea, and K levels were enhanced in the 200 mg/kg extract group. The normal liver and renal markers indicate its ability to improve liver function and the detoxification mechanism. The liver and kidneys are vital organs associated with metabolism, detoxification, and excretion of waste metabolites and end‐products. Therefore, the trivially elevated parameters are due to the continuous consumption of plant extract. The cytochrome P450 is responsible for detoxification through neutralizing toxins and then removal from the body via urination or stool (Esteves et al. 2021). The studies on the toxicity and efficacy of *Hibiscus rosa‐sinensis* reported its safety for consumption. In a study, Wistar albino rats were supplemented with 200 mg/kg ethanolic leaf extract of *Hibiscus rosa‐sinensis* for sub‐acute toxicity. The findings showed that there was no sign of toxicity in animals, and all animals were safe during the study. Thus, the study concluded that 200 mg/kg ethanolic leaf extract of *Hibiscus rosa‐sinensis is* well tolerated with no histopathological and biochemical abnormalities (Mondal et al. 2016). Combining *Hibiscus rosa‐sinensis* flower extract and *Lansium domesticum* fruit extract for 4 weeks proved safe for human eyes and skin as a cosmetic active ingredient (Kilala Tilaar et al. 2018). Diabetic‐induced hepato‐renal damage could be challenging and needs to be addressed appropriately. Aqueous‐methanolic extract of *Hibiscus rosa‐sinensis leaves* (400 mg/kg) significantly reduced glucose levels and alleviated diabetic‐induced hepato‐renal injury (Zaki et al. 2017). In a clinical study, 32 male subjects aged 21–32 years were administered a 500,500 mL *H. sabdariffa* drink for 2 weeks. The results showed that WBCs were reduced, but there was no significant variation in lymphocytes and hepato‐renal biomarkers. They concluded that no hepatotoxicity and kidney damage had been observed in the study (Tazoho et al. 2016).

The oxidative stress markers, WBCs, showed non‐significant values, verifying their safety. However, minor changes were observed in monocytes and basophils. The monocytes were slightly augmented in the 1000 mg/kg powder and 100 mg/kg extract groups. WBCs declined in powder groups while increasing in the 200 mg/kg extract group, and neutrophils were reduced in all supplemented groups. The unsubstantial elevation of WBCs may be due to acute local inflammation. Despite these fluctuations, all values are in the normal range, and the normal values of immune cells indicate the immuno‐modulatory and anti‐inflammatory roles of *Hibiscus rosa‐sinensis* . The studies on *Hibiscus rosa‐sinensis reported* its mixed impact on hematology. In an in vivo study, the impact of *Hibiscus rosa‐sinensis* (40 mg/kg) aqueous and methanolic extracts of leaves on the hematology was evaluated, and it was found that the methanolic extract increased WBCs, lymphocytes, and eosinophils at Day 7, while lymphocytes were reduced at Day 3. Moreover, the aqueous extract augmented lymphocytes on Day 7, whereas it exhibited a non‐significant impact on lymphocytes on Day 3 (Al‐Jarah et al. 2017). In another study, the acute and sub‐acute oral toxicity of methanolic extract from leaves of *Hibiscus rosa‐sinensis* in mice was assessed. The study demonstrated that 2000 mg/kg of extract in acute toxicity showed no toxicity or mortality in any animal. However, in the sub‐acute phase, animals revealed elevated biochemical markers, histological aberrations, and hematological variations at 800 mg/kg extract but did not show any sign of toxic effects at 400 mg/kg in the sub‐acute phase. They concluded that LD50 was > 2000 mg/kg, but 400 mg/kg was considered safe (Nath and Yadav 2014). The impact on hematology and other parameters is due to high doses. The histopathological examination revealed no major change or any injury in hepatocytes. Hepatic parenchyma in all observed groups exhibited normal architectural structure with no prominent degeneration or inflammatory response. Hepatocytes were coordinated with prominent centrally placed nuclei without signs of steatosis or necrosis. Our findings are supported by the studies of Mondal et al. (2016) and Tazoho et al. (2016), as they reported no sign of toxicity and histopathological changes in rats and humans when supplemented with 200 mg/kg ethanolic leaves extract of *Hibiscus rosa‐sinensis* and 500 mL *H. sabdariffa* drink, respectively.

## 5 Conclusion

The study revealed that *Hibiscus rosa‐sinensis* contains diverse bioactive compounds and functional groups identified by GC–MS and FTIR spectroscopy analysis. Ethyl esters are dominant compounds, with O–H, –COOH, C≡N, C=O, and C–H as functional groups. Toxicological evaluations demonstrated that both powder and extract forms were well tolerated in animals, with no significant adverse effects observed in biochemical, hematological, or histopathological parameters. Although slight changes were noted in the differential cell count at higher doses (1000 mg/kg powder and 200 mg/kg extract), these were deemed not to be harmful. Overall, the findings support the safe use of *Hibiscus rosa‐sinensis* as a supplement.

## Author Contributions

**Hassan Raza:** conceptualization (equal), formal analysis (equal), writingoriginal draft preparation (equal). **Muhammad Tauseef Sultan:** conceptualization (equal), supervision (equal), project administration (equal). **Ahmad Mujtaba Noman:** writing‐review and editing (equal), visualization (equal). **Ali Imran:** validation (equal). **Sheraz Ahmed Bhatti:** methodology (equal), software (equal). **Iqra Baig:** data curation (equal). **Muhammad Abdul Rahim:** writing‐review and editing (equal), visualization (equal). **Meshari A. Alsuwat:** visualization (equal), funding acquisition (equal). **Mohamed Fawzy Ramadan:** writing‐review and editing (equal), funding acquisition (equal). **Eliasse Zongo:** visualization (equal), funding acquisition (equal). All authors have read and agreed to the published version of the manuscript.

## Funding

This research was funded by Taif University, Taif, Saudi Arabia project No. (TU‐DSPP‐2025‐02).

## Ethics Statement

All steps of this experiment were used in accord with Internationally Accepted Guidelines for Animal Research as prescribed by the Declaration of Helsinki. All animal procedures were approved by the Research Ethics Committee of the Bahauddin Zakariya University, Multan, Punjab, Pakistan. Members of the Institutional Review Board, Bahauddin Zakariya University, Multan, checked the research plan and issued a letter signed by the members of the Review Board (REC No. 014, 05/04/2024).

## Consent

All the authors in the study agree to the work and give their consent for the publication.

## Conflicts of Interest

The authors declare no conflicts of interest.

## Data Availability

The data of this study will be made available on request from the corresponding author.
